# Detecting QTLs and putative candidate genes involved in budbreak and flowering time in an apple multiparental population

**DOI:** 10.1093/jxb/erw130

**Published:** 2016-03-31

**Authors:** Alix Allard, Marco C.A.M. Bink, Sébastien Martinez, Jean-Jacques Kelner, Jean-Michel Legave, Mario di Guardo, Erica A. Di Pierro, François Laurens, Eric W. van de Weg, Evelyne Costes

**Affiliations:** ^1^Institut National de la Recherche Agronomique (INRA), UMR 1334, AGAP CIRAD-INRA-Montpellier SupAgro, F-34398 Montpellier, France; ^2^Montpellier SupAgro, UMR 1334, AGAP CIRAD-INRA-Montpellier SupAgro, F-34398 Montpellier, France; ^3^Biometris, Wageningen University and Research centre, Droevendaalsesteeg 1, PO Box 16, 6700AA, Wageningen, The Netherlands; ^4^Research and Innovation Centre, Fondazione Edmund Mach, San Michele all’Adige, Trento, Italy; ^5^Wageningen UR Plant Breeding, Wageningen University and Research Centre, Droevendaalsesteeg 1, PO Box 16, 6700AA, Wageningen, The Netherlands; ^6^Department of Biosciences, University of Milan, Via Celoria 26, 20133 Milan, Italy; ^7^INRA, UMR1345, Institut de Recherche en Horticulture et Semences IRHS, INRA, Agrocampus-Ouest, Université d’Angers, SFR 4207 QUASAV, F-49071 Beaucouzé, France

**Keywords:** Climate change, *DAM* genes, dormancy, flowering genes, *Malus×domestica* (Borkh), pedigree-based analysis, phenology, QTL.

## Abstract

In temperate trees, growth resumption in spring time results from chilling and heat requirements, and is an adaptive trait under global warming. Here, the genetic determinism of budbreak and flowering time was deciphered using five related full-sib apple families. Both traits were observed over 3 years and two sites and expressed in calendar and degree-days. Best linear unbiased predictors of genotypic effect or interaction with climatic year were extracted from mixed linear models and used for quantitative trait locus (QTL) mapping, performed with an integrated genetic map containing 6849 single nucleotide polymorphisms (SNPs), grouped into haplotypes, and with a Bayesian pedigree-based analysis. Four major regions, on linkage group (LG) 7, LG10, LG12, and LG9, the latter being the most stable across families, sites, and years, explained 5.6–21.3% of trait variance. Co-localizations for traits in calendar days or growing degree hours (GDH) suggested common genetic determinism for chilling and heating requirements. Homologs of two major flowering genes, *AGL24* and *FT*, were predicted close to LG9 and LG12 QTLs, respectively, whereas *Dormancy Associated MADs-box (DAM*) genes were near additional QTLs on LG8 and LG15. This suggests that chilling perception mechanisms could be common among perennial and annual plants. Progenitors with favorable alleles depending on trait and LG were identified and could benefit new breeding strategies for apple adaptation to temperature increase.

## Highlight

QTLs and candidate genes for the regulation of budbreak and flowering time reveal new hypotheses on temperature perception in growth resumption at spring time in apple.

## Introduction

Global warming has an impact on tree phenology, and rising temperatures in late autumn and winter can lead to problematic conditions regarding bud dormancy release. Dormancy is a mechanism developed by perennial plants growing in temperate climates to overcome periods of cold temperatures and to protect buds from frost. Dormancy has been described as being composed of three states: (i) paradormancy, which is regulated by physiological factors outside the bud, such as apical dominance; (ii) endodormancy, which is regulated by physiological factors inside the bud and during which the perception of chilling temperatures is active; and (iii) ecodormancy, which is regulated by environmental factors, especially temperature (Lang *et al.*, 1987). Dormancy release is mainly driven by exposure to chilling temperatures (Heide and Prestrud, 2005; Schoot *et al.*, 2013), whereas growth rate is driven by warm temperatures (Wigge, 2013). The chilling requirement (CR) corresponds to the amount of exposure time to cold temperatures required for dormancy release and is usually expressed in chilling hours (Anderson and Richardson, 1986). The heat requirement (HR) represents the amount of exposure time to warm temperatures required to reach a particular physiological stage, generally budbreak or flowering time. Both stages are considered to be the result of the cumulated time necessary to satisfy CR and HR.

CR and HR have been extensively studied in cultivated trees (Citadin *et al.*, 2001; Egea *et al.*, 2003; Ruiz *et al.*, 2007; Alburquerque *et al.*, 2008; Guo *et al.*, 2014). A lack of synchronization between flowering and temperature causes bud frost, extended flowering time, poor pollination, and fruit setting (Atkinson *et al.*, 2013). In the Rosaceae family, it has been established that at the species level and in a fixed environment, CR has more influence on flowering date than HR for *Prunus* and apple (Egea *et al.*, 2003; Ruiz *et al.*, 2007; Alburquerque *et al.*, 2008; Fan *et al.*, 2010; Celton *et al.*, 2011). Moreover, some interdependency between CR and HR has been suggested: a lack of chilling causes an extended need for warm temperatures, probably due to a residual effect of dormancy, whereas extended exposure to chilling temperatures leads to a reduced HR (Ruiz *et al.*, 2007). Models with an overlap between CR and HR fulfillment have been proposed for prediction of the dates of phenological stages in order to account for a possible influence of cold temperature after dormancy release (Pope *et al.*, 2014). For a given cultivar, the number of days required to fulfill CR and HR depends on the climatic region, in particular in subtropical and Mediterranean regions where dormancy tends to be released later (Legave *et al.*, 2012). The impact of increasing temperatures on phenology has been extensively studied in different climates: temperate (Howe *et al.*, 2000; Wolfe *et al.*, 2005; Legave *et al.*, 2009; Fujisawa and Kobayashi, 2010), and Mediterranean and subtropical (Gordo and Sanz, 2009; Legave *et al.*, 2009; Grab and Craparo, 2011; Darbyshire *et al.*, 2013; Ghrab *et al.*, 2014). In the context of global warming, the trend tends towards an earlier growth resumption, which would be due to a reduced time for HR fulfillment (Wolfe *et al.*, 2005; Gordo and Sanz, 2009; Fujisawa and Kobayashi, 2010; Grab and Craparo, 2011; Legave *et al.*, 2012). In the coming decades, climate models predict a reduction in chill accumulation that could be an advantage for countries at high latitudes such as Norway (Campoy *et al.*, 2011), since it would still allow chill accumulation while avoiding spring frost (Luedeling, 2012). However, in warmer regions, a major loss of chill accumulation is expected (Campoy *et al.*, 2011).

The concept of common physiological mechanisms between flowering promoted by vernalization and dormancy has been strongly reinforced since Chouard’s work in 1960 (Horvath *et al.*, 2010; Rios *et al.*, 2014). In fruit trees, the comprehension of molecular control of dormancy has been improved by the study of an evergrowing mutant (*evg*) in peach in which six *DAM* genes have been identified (Bielenberg *et al.*, 2008; Jiménez *et al.*, 2010). These *DAM* genes (especially *ppDAM5* and *ppDAM6*) have been shown to belong to the *SHORT VEGETATIVE PHASE* (*SVP*)/*AGAMOUS-LIKE 24* (*AGL24*) gene family in *Arabidopsis thaliana* (Yamane *et al.*, 2011; Wells *et al.*, 2015), whose members control flowering and are up-regulated by vernalization (Michaels *et al.*, 2003). Their orthologs in peach and Japanese pear have been shown to be involved in dormancy establishment and release, regulation of chilling, and heat perception, and could also be part of a down-regulation system for bud development (Jiménez *et al.*, 2010; Saito *et al.*, 2013). In apple, four *MdDAM* genes have been identified (Mimida *et al.*, 2015). *MdDAMa* (MDP00003222567) expression peaked at dormancy establishment, and gradually decreased under the influence of chilling temperatures until dormancy release (Falavigna *et al.*, 2013). Different chilling treatments have revealed other differentially expressed genes with a putative role in dormancy regulation, especially a FLOWERING LOCUS C-like gene (*FLC*-like) located at the top of linkage group (LG) 9 (Porto *et al.*, 2015). Other regulatory genes belonging to the *MADS-box* family have also been found to be related to dormancy events. In particular, *FLOWERING LOCUS T* (*FT*) homologs would be involved in the transition between reproductive and vegetative growth, growth cessation in response to short photoperiod and winter temperatures, dormancy requirement, and promotion of early flowering in trees (Böhlenius *et al.*, 2006; Kotoda *et al.*, 2010; Tränkner *et al.*, 2010; Hsu *et al.*, 2011; Srinivasan *et al.*, 2012). Since budbreak and flowering time have been shown to be heritable traits (Labuschagne *et al.*, 2002), genetic breeding could be a way to adapt fruit trees to a changing environment. Several quantitiative trait loci (QTLs) were detected with bi-parental families on *Prunus* and *Malus* species. On *Prunus*, a co-localization between a flowering date and CR QTL has confirmed the hypothesis of a common genetic determinism between those traits and the high complexity of their genetic control, with QTLs detected on almost all the LGs (Dirlewanger *et al.*, 2012). In apple, the top of LG9 has been identified as an important QTL region in two progeny (Dyk *et al.*, 2010; Celton *et al.*, 2011), and complex genetic control has been revealed with several QTLs on different LGs, depending on the progeny (Liebhard *et al.*, 2003; Segura *et al.*, 2007; Celton *et al.*, 2011). However, single bi-parental analyses did not provide results that were transferable to other progeny, except for LG9 in apple. This is a problem common to many species that can be addressed by QTL mapping on multiparental populations which extend the genetic diversity and allow the comparison of allele performance in different genetic backgrounds (Pauly *et al.*, 2012). This strategy has been shown to increase QTL detection power, accuracy of QTL positions, and robustness of estimation of QTL effects (Bink *et al.*, 2002; Blanc *et al.*, 2006; Liu *et al.*, 2012). However, false-positive QTLs can be detected with such populations due to relationships between individuals. Pedigree-based analysis (PBA) overcame this issue by taking into account relationships between individuals using the concept of identity by descent (IBD), which combines information from both pedigree and markers (Luan *et al.*, 2012). In addition, considering marker data for common ancestors makes it possible to trace the source of favorable alleles.

In this study, our first aim was to map QTLs linked to bud phenology and to distinguish those linked to CR or HR through a multifamily and pedigree-based analysis. A second aim was to identify underlying candidate genes, paying special attention to related *DAM* genes. The third aim of the study was to identify founders, parents, or individuals with interesting genotypes and to trace their transmission along the pedigree. For these purposes, several variables that represented variation due to CR or HR or both were studied. Five families and their genetic relationships were considered in a pedigree-based analysis. This study revealed four important loci on LG7, LG9, LG10, and LG12, and two minor loci on LG8 and LG15. For the first time in apple, candidate genes previously described as being involved in chill perception and flowering, namely *DAM* genes, homologs to *AGL24*, *FT*, and *FLC* in *A. thaliana*, were mapped under four QTLs. Moreover, progenitors with favorable alleles were identified that could open up new perspectives for breeding.

## Materials and methods

### Plant material

**Fig. 1.**
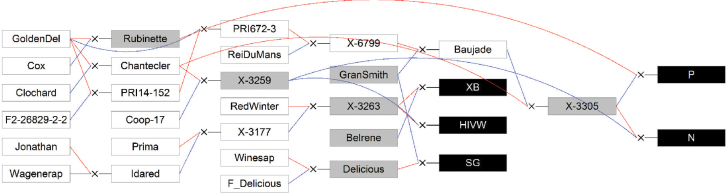
Genetic relationships between the five full-sib families. The full-sib families are represented by black boxes; the parents by gray boxes; the founders and other members of the pedigree by white boxes. Blue and red lines link the father and mother, respectively, to its progeny. GoldenDel, Golden Delicious; ReiDuMans, Reinette du Mans; Wagenerap, Wagenerapfel; for XB, HIVW, SG, N and P (see abbreviations list for meaning).(This figure is available in colour at *JXB* online.)

Five related full-sib families were considered. Two families were grown at INRA’s Diascope experimental unit in Montpellier (co-ordinates: 43°36'35''N; 3°58'50''E) and three at INRA’s Angers experimental station (co-ordinates: 47°29'7.656N; 0°36'47.646). The Montpellier families were derived from a cross between a color mutant of ‘Delicious’, ‘Starkrimson’, and ‘Granny Smith’, and between X-3263 and ‘Belrene’ (Fig. 1). They were designated SG and XB, and were composed of 115 and 58 progeny, respectively. SG progeny were repeated twice. For both families, trees were grafted on Pajam I apple rootstock; SG was planted in 2007 and XB in 2005. Trees were grown with minimal training and pruning. The Angers families were derived from crosses between X-3263 and X-3259, X-3259 and X-3305, and X-3305 and ‘Rubinette’, and were designated HIVW, N, and P, respectively (Fig. 1). They were composed of 171, 42, and 45 individuals, respectively, each with a single replicate per individual. The trees were trained in the vertical axis with an annual manual thinning with one fruit per inflorescence. At both sites, pest and disease management was performed consistently with professional practices.

The families were interconnected through a complex pedigree (Fig. 1). X-3263 is the parent of both XB and HIVW, X-3259 is the parent of both N and HIVW, X-3305 is the parent of both N and P, and ‘Chantecler’ is the mother of both X-3305 and X-3259. Moreover, ‘Golden Delicious’ is the founder of several parents, ‘Chantecler’, ‘PRI672-3’, ‘PRI14-126’, and ‘Rubinette’. ‘Granny Smith’ is the parent of ‘Baujade’ and the SG family. Compared with the low chilling cultivar ‘Anna’ and the high chilling cultivar ‘Golden Delicious’ (requiring 300 and 1000 chilling hours, respectively) (Labuschagne *et al.*, 2002), ‘Granny Smith’ and ‘Delicious’ are considered as relatively low chilling cultivars since they require 600 and 700 chilling hours, respectively (http://www.orangepippin.com). Parents X-3305, X-3259 and X-3263 were selected by INRA for their high fruit quality and scab resistance.

### Phenotypic assessment

Two phenological stages were observed: budbreak (BB) and beginning of flowering (BF) (Fleckinger, 1964). Each stage was evaluated at the tree scale, and the stage was considered to be reached when 50% of the buds reached it. To avoid missing a stage, the assessment was made three times a week from mid-March to May. Progeny were phenotyped in three consecutive years, 2012, 2013, and 2014.

### Year characterization

For each year and site, the dormancy release date and flowering date were predicted for the reference high CR cultivar ‘Golden Delicious’, for which CR and HR are known, using the model of Legave *et al.* (2012). This model uses a sequential approach where (i) dormancy release is predicted when CR is fulfilled; (ii) heat perception starts once CR is fulfilled; and (iii) flowering date is predicted when HR is fulfilled. The input data of this model are the mean daily temperatures of the corresponding sites and years.

### Phenological stage modeling

Since BB and BF dates result from fulfillment of both CR and HR, we intended to differentiate their effect by using two different units: calendar days (CD) and growing degree hours (GDH). Budbreak in calendar days (BB_CD) was calculated from 1^st^ of January, since most models consider a fixed date to start the calculation of chilling unit accumulation whatever the variety (Bidabé, 1967). This variable was considered as representative of variations in time to fulfill both CR and HR. Budbreak date in growing degree hours (BB_GDH) was calculated between the date of dormancy release estimated for Golden Delicious by the model of Legave *et al.* (2012) and the observed budbreak date for each genotype. Growing degree hours accounted for temperatures above a certain threshold and in a certain range, following the classical method used in apple to quantify the amount of warm temperature cumulated (Anderson and Richardson, 1986):

$$\begin{array}{l} \text{If TH}<\text{TU},\;GDH_{1}=\frac{F\times (TB-TU)}{2}\\ \;\times \left[1+\cos(\pi +\frac{\pi \times (TH-TB)}{TU-TB})\right] \end{array}$$ (1)

$$\text{If TH}>\text{TU},\;GDH_{2}=(TB-TU)\times \left[1+\cos(\frac{\pi }{2}+\frac{\frac{\pi }{2(TH-TU)}}{TC-TB})\right]$$ (2)

where GDH is the accumulation of growing degree hours during 1h; TH is the hourly temperature; TB is the base temperature (4 °C); TU is the optimum temperature (25 °C); and TC is the critical temperature (36 °C)

The threshold temperatures also correspond to values generally used for fruit trees, according to Anderson and Richardson (1986). Finally, the heat accumulation was also quantified between the dates of budbreak and the beginning of flowering (Delta_GDH; see abbreviation list). BF, which was highly correlated with BB_CD, was not considered in later analyses.

### Phenotype modeling

Linear models were used to assess the effect of factors, Year, site, genotype, and the genotype×year interaction for each trait, BB_CD, BB_GDH and Delta_GDH:

$$y=X_{\text{1}}\beta_{\text{1}}+X_{\text{2}}\beta_{\text{2}}+Z_{\text{1}}u_{\text{1}}+Z_{\text{2}}u_{\text{2}}+e$$ (3)

where *y* is the variable. *X*_1_ is a matrix of dimension ‘number of individual trees’×2, associated with the site fixed effect. *X*_2_ is a matrix of dimension ‘number of trees’×3, associated with the year fixed effect. β_1_ and β_2_ are vectors of length 2 and 3, respectively, associated with site and year fixed effects, respectively. *Z*_1_ is a matrix of dimension ‘number of trees’×‘number of genotypes’, associated with the genotype random effect. *Z*_2_ is a matrix of dimension ‘number of trees’×(‘number of genotypes’×3), associated with the genotype×year interaction random effect. *u*_1_ and *u*_2_ are vectors of length, ‘number of genotypes’ and ‘number of genotypes’×3, respectively, associated with genotype and interaction year×genotype random effects, respectively. Finally, *e* is the vector of residual variance.

Preliminary analysis revealed that variances differed significantly between years, and correlations between years were positive. The heterogeneous variances per year were modeled in the residual term and the interaction factor, and correlations between years were accounted for by fitting an unstructured variance–covariance matrix on the residual term in the linear mixed models. All these models were solved with ASReml-R software (Gilmour *et al.*, 1995).

The mixed model yielded the best unbiased linear predictors (BLUPs) for genotype and genotype×year interaction random factors for BB_CD, BB_GDH, and Delta_GDH. These BLUP values were used for QTL mapping. For each trait, a QTL analysis was performed for the genotypic BLUPs (G_*j*_) and for the interaction BLUPs (G_*j*_×Y_*k*_), resulting in a total of 12 variables. The mixed models also yielded estimates for the variance components, and the mean broad sense heritability was calculated as:

$$h^{2}=\frac{\sigma_{G}^{2}}{\sigma_{G}^{2}+\frac{\sigma_{G\times Y}^{2}}{k}+\text{​}\frac{\sigma_{\varepsilon }^{2}}{n}}$$ (4)

where $\sigma_{G}^{2}$ is the variance of genotype effect, $\sigma_{G\times Y}^{2}$ is the variance of genotype×year interaction effect, $\sigma_{\varepsilon }^{2}$ is the variance of the residual term, *k* is the number of years, and *n* is the total number of observations.

It was not possible to assess the interaction between genotypes and sites because each family was present in one site only. Consequently, in order to assess the stability of QTLs across sites, each trait was analyzed with three data sets: the full set containing the two sites (multisite analysis); the two complementary subsets of two Montpellier families (Mtp analysis); and three Angers families (Ang analysis; see abbreviation list).

### Single nucleotide polymorphim (SNP) marker data

The five full-sib families and their progenitors (when available; see Fig. 1) were genotyped with the Infinium^®^ 20K SNP array (Bianco *et al.*, 2014) at the Fondazione Edmund Mach according to the procedures described by Chagné *et al.* (2012) and Antanaviciute *et al.* (2012). A total of 6849 of the SNPs were used in this study, after (i) having passed the Excel-based forerunner of the ASSIsT pipeline (Di Guardo *et al.*, 2015), for 27 full-sib families; (ii) having shown robust performance over the pedigrees of these families (E.W. van de Weg *et al*., unpublished); and (iii) having shown robust performance on the current germplasm. For the latter, genotype score consistency was assessed between individuals and their progeny and parents. When errors systematically arose for a marker, it was removed from the data set. The recombination pattern was checked in order to identify spurious double recombination events, and problematic markers were removed. The genetic positions of these SNP were taken from a pre-runner of the consensus genetic map based on 21 of the above 27 families. The marker order of this map was validated through co-segregation patterns across all 27 families and their pedigrees (E.W. van de Weg, personal communication). Next, sets of SNPs were integrated into haploblocks. The genetic map was then divided into successive 1 cM segments and the SNPs of each segment were assigned to a single haploblock. Haplotypes were composed using FlexQTL™ and PediHaplotyper software (Voorrips *et al.*, 2016). At the marker level, double recombination occurred in 5.2% of the LGs, whereas, at the haplotype level, they occurred in 0.5% of the LGs. Since they were spread over the genome and progeny, they were not considered as problematic. Haploblocks along the genome were highly informative, considering the proportion of informative meioses (Supplementary Fig. S1 at *JXB* online).

### Bayesian QTL mapping methodology

The 12 variables, namely the genotypic BLUP and three genotype×year interaction BLUPs for BB_CD, BB_GDH, and Delta_GDH were analyzed using a linear model that comprised an intercept μ, the regressions on the QTL covariates *a*, and a model residual *e*, as:

y=1μ+Wa+e (5)

where *W* is the design matrix for the QTL effects. The Bayesian modeling including the number of QTLs is a random variable and, consequently, the number of columns in *W* is not fixed (Bink *et al.*, 2008). A bi-allelic model is assigned to a QTL with alleles denoted by *Q* and *q*, with only additive effects modeled, and the covariate values of [*QQ*, *Qq*, *qq*] are equal to [1, 0, −1]. The frequency of the allele Q among founder individuals is denoted by *f*_*a*_, and the linkage map positions of the QTL are given by vector λ. The QTL genotypes of individuals are *a priori* unknown, and modeling is based on the independent assignment of alleles to founders and segregation indicators to trace transmission from parents to offspring (Bink *et al.*, 2002; Thompson *et al.*, 2008). Our Bayesian modeling assigns uniform priors to the variables μ and λ, while assigning normal priors to the vectors *a* and *e* in Equation 5; namely $a\approx N(0,\sigma_{a}^{2})$ and $e\approx N(0,\sigma_{e}^{2})$. The variables $\sigma_{a}^{2}$ and $\sigma_{e}^{2}$are the per-QTL explained variance and the residual variance, with priors being inverse Gamma distributions (Bink *et al.*, 2008). The number of QTLs was assigned a Poisson prior where different values (i.e. 5, 10) were used to assess the sensitivity of posterior inference to the prior assumptions. Only results for a prior mean of five are reported since the other values yield similar results and inferences.

Markov chain Monte Carlo (MCMC) simulation, as implemented in FlexQTL™ software (Bink *et al.*, 2014), was applied to obtain samples from the joint posterior distribution of the model parameters:

$$f(\mu ,\alpha ,\lambda ,\sigma_{a}^{2},\sigma_{e}^{2}|y)$$ (6)

The MCMC algorithms have been previously described (Bink *et al.*, 2008) and are omitted here. The Monte Carlo accuracy was monitored and the length of the simulation chains was required to be equivalent to at least 100 effective chain samples. The required lengths of the Markov chain simulations varied among traits but never exceeded 500 000 iterations. A total of 1000 samples were stored for each simulation and are thus available for statistical inference. The inference on the number of QTLs was based on a pairwise comparison of models differing from each other by one QTL, by taking twice the natural log of Bayes factors (Kass and Raftery, 1995), denoted as 2×lnBF. Values for 2×lnBF that are >2, 5, and 10 indicate positive, strong, and decisive evidence, respectively, favoring the largest QTL model. Similar to Bink *et al.* (2014), the inferences on QTL positions are based on posterior QTL intensities, and the inference on QTL contributions are based on the posterior mean estimates of the QTL effect sizes. Posterior probabilities of QTL genotypes were also estimated, and the same thresholds as in Bink *et al.* (2014) were used.

### Identification of genes underlying QTLs

For each QTL region, flanking markers were identified and localized on the first and the third version of the apple genome (kindly provided by the Fondazione Edmund Mach, now publicly available at https://www.rosaceae.org/) because gene prediction was performed on the first version only. The list of genes under the QTL interval was screened on the first version in order to identify genes related to flowering time, *A. thaliana* flowering pathway, temperature response, dormancy, and vernalization. Genes involved in the regulation of cell cycles or associated with plant hormones that have been previously identified in Celton *et al.* (2011) were not considered in the present study. The presence of candidate genes in the same interval and the corresponding scaffold was checked on the third version.

## Results

### Year and site characterization

**Fig. 2.**
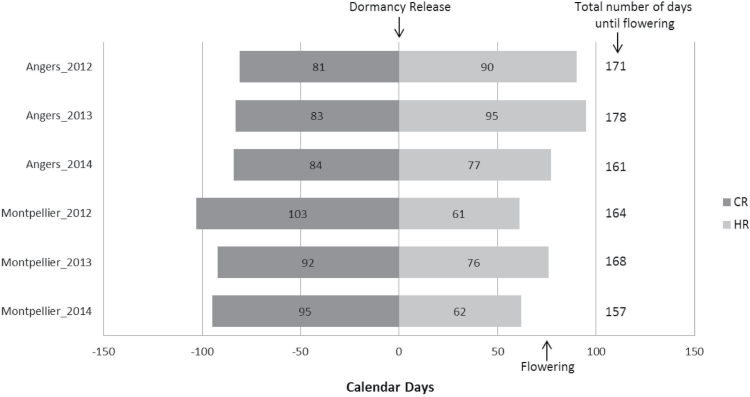
Characterization of climatic years (2012, 2013, and 2014) and sites (Angers and Montpellier) by observed flowering dates and estimation of dormancy release date of cultivar ‘Golden Delicious’ with the model of Legave *et al.* (2012). The dark gray blocks represent the estimated number of days before dormancy release, and the light gray blocks the number of days between dormancy release and observed flowering dates.

The predicted dormancy release dates and the observed flowering dates for ‘Golden Delicious’ in each year and at both sites provided insights into differences in climatic conditions (Fig. 2; Supplementary Figs S2–S4 for daily temperatures). At both sites, flowering time happened later in 2013 and earlier in 2014 than in the other year. For Angers, the dormancy release date predicted for ‘Golden Delicious’ seemed to be stable, with a maximum of a 3 d difference between years, whereas a larger variance was observed in the number of days to fulfill the HR, which varied between 77 d and 95 d. Therefore, in Angers climatic conditions, CR was easily met and the variation in flowering date would mainly be due to the variation in time to fulfill HR. In Montpellier, the dormancy release date varied between years, up to 11 d between the earliest (2013) and the latest (2012) date. The number of days to fulfill HR also varied by 15 d between 2012 (lowest value) and 2013 (highest value). Therefore, in Montpellier, the variation in flowering date was due to the variation in time to fulfill both CR and HR.

### Phenotypes and heritabilities

**Fig. 3.**
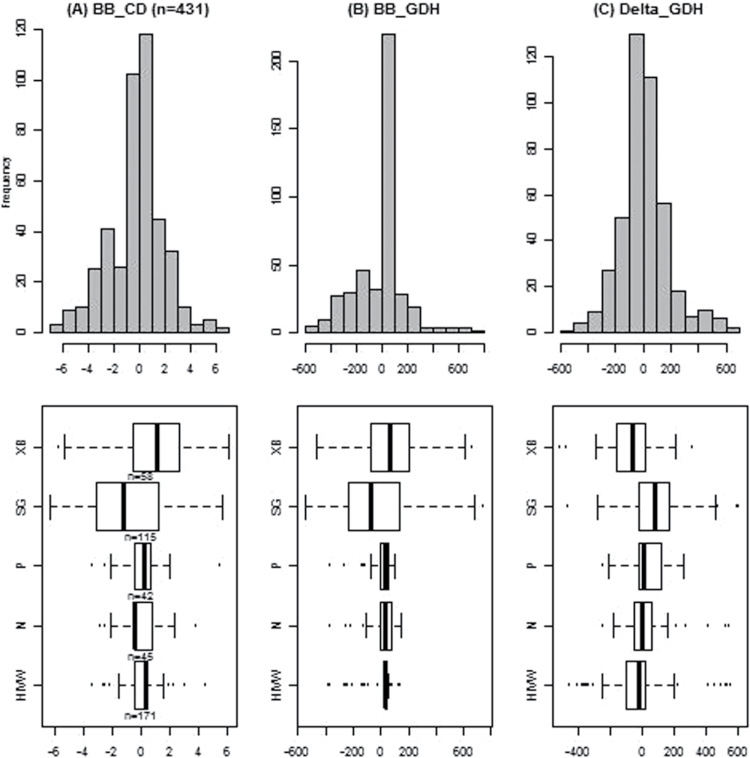
Upper part: histograms of genotypic best linear unbiased predictor (BLUP) for BB_CD (A), in BB_GDH (B), and Delta_GDH (C). Lower part: boxplots for each full-sib family (XB, SG, P, N, HIVW) and for BB_CD (left), BB_GDH (middle), and Delta_GDH (right). For each boxplot, the bold line represents the median, the extremities of the box represent the first and third quartile, from left to right, respectively, and the whiskers represent extreme values. See abbreviation list for family abbreviations.

Phenotype distributions differed between sites: the families from Montpellier (XB and SG) had a wider distribution than the families from Angers, where distributions of BB_GDH were particularly narrow for P, N, and HIVW (Fig. 3). Heritabilities were 0.87, 0.38, and 0.41 for BB_CD, BB_GDH, and Delta_GDH, respectively. Correlation tests revealed a significant and positive correlation between BB_CD and BB_GDH (*r* ^2^=0.89) (Supplementary Fig. S5), while Delta_GDH was not correlated to other variables.

### QTL discovery for genotypic BLUPs

**Fig. 4.**
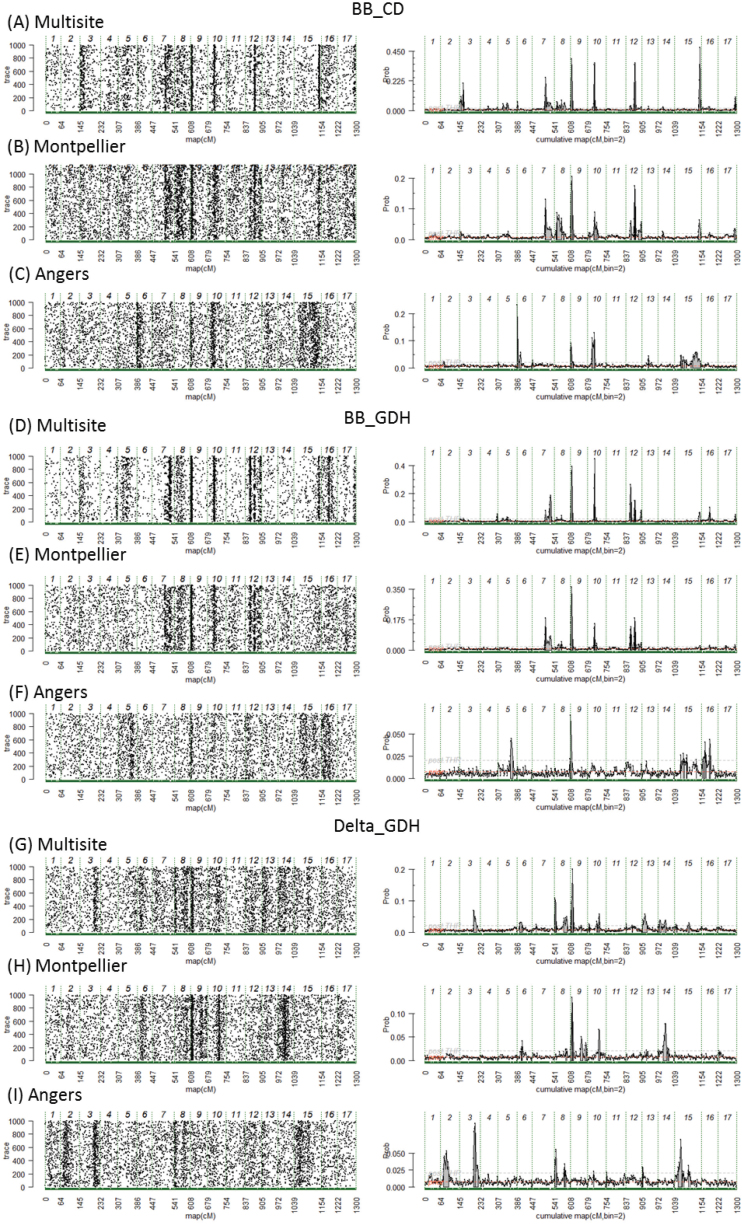
Trace plots (left) and posterior probability of QTL positions (right) along the genome for genotypic BLUPs, for trait BB_CD in the (a) multisite, (b) Montpellier, and (c) Angers analyses, for trait BB_GDH in the (d) multisite, (e) Montpellier, and (f) Angers analyses, and for trait Delta_GDH in the (g) multisite, (h) Montpellier, and (i) Angers analyses. The beginning and the end of the chromosomes are represented by vertical dashed lines. The solid gray areas on the right side correspond to regions with positive evidence (2×lnBF_10_ >2) for the presence of QTLs.

Four regions considered as major were identified on LG7, LG9, LG10, and LG12 because of their high 2×lnBF values, their stability across sites and variables (Fig. 4; Table 1), and, in some cases, because of the presence of a candidate gene in the QTL interval. The most stable of these regions was located at the top of LG9 (Fig. 4A–I; Table 1). It gave a QTL signal for BB_CD, BB_GDH, and Delta_GDH, and for the multisite as well as for site-specific analyses, even though the signal was not significant in Angers. This QTL explained from 11.5% to 18.2% of the trait variance (Table 1). In this region, MDP0000126259, a homolog of *FLOWERING LOCUS C* (*FLC*-like), was predicted in both the first and third versions of the apple genome (Table 2). Moreover, MDP0000143531, a homolog of *AGL24* in *A. thaliana*, was predicted below the lower end of the QTL.

**Table 1.** Parameters associated with the genotypic BLUP QTLs The first column indicates the type of analysis, the second one the trait concerned, and the following columns indicate the LG where the QTL is located, the 2×lnBF value at the LG scale for a 1 QTL over a 0 QTL model, the 2×lnBF value at the local scale, the position of the QTL region in cM, the additive QTL effect, the frequency of the positive allele, its variance, and its percentage of variance explained. The 2×lnBF values for multi-QTL models were not presented because none of them passed the significance threshold. The QTLs with a 2×lnBF at the LG scale higher than 5 are in bold.

**Analysis**	**trait**	**LG**	**2×lnBF_LG**	**max 2×lnBF_loc**	**Pos (cM**)	**add**	**fq**	**var**	**%var**
Multisite	BB_CD	3	4.8	7.0	1–21	0.77	0.40	0.28	4.6
		**7**	**9.4**	**7.5**	**54–88**	**1.17**	**0.51**	**0.69**	**11.1**
		8	3.8	4.8	11–47	0.98	0.71	0.39	6.3
		**9**	**29.9**	**8.8**	**0–11**	**1.76**	**0.21**	**1.03**	**16.6**
		**10**	**10.9**	**8.6**	**22–35**	**0.92**	**0.65**	**0.35**	**5.6**
		**12**	**29.9**	**8.6**	**37–43**	**2.05**	**0.80**	**1.32**	**21.3**
		**15**	**9.1**	**9.5**	**105–111**	**0.99**	**0.70**	**0.42**	**6.7**
	BB_GDH	**7**	**29.4**	**6.3**	**56–84**	**189.8**	**0.24**	**13 070**	**21.3**
		**9**	**30.1**	**8.8**	**4–10**	**193.0**	**0.82**	**11 139**	**18.2**
		**10**	**9.4**	**9.2**	**28–34**	**106.4**	**0.53**	**5640**	**9.2**
		**12**	**29.6**	**7.3**	**19–43**	**182.0**	**0.67**	**14 726**	**24.0**
	delta_GDH	8	3.5	5.5	1–9	59.4	0.64	1628	5.2
		9	4.4	6.9	4–14	98.9	0.24	3572	11.5
Mtp	BB_CD	7	4.0	5.9	56–84	1.45	0.45	1.0	10.4
		**8**	**5.1**	**5.0**	**9–31**	**1.15**	**0.51**	**0.7**	**6.6**
		**9**	**4.8**	**7.0**	**2–14**	**1.63**	**0.51**	**1.3**	**13.3**
		10	3.0	5.1	26–40	1.14	0.60	0.6	6.2
		**12**	**6.1**	**6.6**	**19–67**	**1.88**	**0.62**	**1.7**	**16.7**
	BB_GDH	**7**	**7.2**	**6.8**	**54–86**	**153.3**	**0.47**	**11 723**	**12.0**
		**9**	**10.2**	**8.6**	**2–12**	**183.7**	**0.49**	**16 858**	**17.2**
		10	3.2	6.3	26–40	108.9	0.59	5722	5.8
		**12**	**10.4**	**6.7**	**19–43**	**189.0**	**0.56**	**17 609**	**18.0**
	delta_GDH	9	3.6	5.5	2–14	107.4	0.40	5538	15.9
		14	3.4	4.9	21–37	89.7	0.46	3993	11.5
An	BB_CD	6	4.5	8.0	1–15	0.55	0.59	0.1	6.6
		10	4.3	6.4	16–34	0.65	0.50	0.2	9.5
		15	4.0	4.4	77–109	0.70	0.65	0.2	10.0
	BB_GDH	no QTL							
	delta_GDH	2	2.5	4.0	13–35	60.9	0.52	1851	6.9
		3	2.7	5.2	59–79	58.8	0.48	1724	6.4

**Table 2.** Candidate gene information underlying QTLs The first five columns contain information about the QTL: LG, position in cM, flanking markers, and their physical position on the first and third versions of the apple genome. Columns 6–9 contain information about candidate genes underlying the QTLs, or that are located nearby: their name and annotation on the apple genome and/or their homolog in *Arabidopsis thaliana*, their position on the first version of the genome, the related literature reference, and their position on the third version when available.

**LG**	**QTL position (cM**)	**QTL flanking markers**	**Flanking marker position**		**Candidate gene**	**Candidate gene position** **Apple genome v1**	**Reference**	**Candidate gene position** **Apple genome v3**
			**Apple genome v1**	**Apple genome v3**				
9	Start: 0 End: 10	SNP_FB_0771340 SNP_FB_0774491	Chr9:0.8 Chr9:2.4	Chr9: 1.50 Chr9: 3.07	MDP0000143531 (*AGL24*)	Start: chr9: 4.52 End: chr9: 4.54		MDC021673.64 Scaffold300219 Start: chr9:5.21 End: chr9:5.22
					MDP0000126259 (*FLC-like*)	Start: chr9: 0.695 End: chr9: 0.697	Porto *et al.* (2015)	
12	Start: 43 End: 67	SNP_FB_0141443 SNP_FB_0155354	Chr12:23.5 Chr12:31.2	Chr12: 28.2 Chr12: 37.4	MDP0000132050 (*MdFT1*)	Start: chr12: 31.58 End: chr12: 31.58	Guitton *et al.* (2011)	MDC021142.191 Scaffold300114 Start: chr12: 37.86 End: chr12: 37.87
15	Start: 105 End: 111	SNP_FB_0914634 SNP_FB_0327408	Chr15: 39.1 Chr15: 43.0	Chr15: 48.5 Chr15:53.1	MDP0000322567 (*MdDAMa*, *AGL24*)	MDC020688.360 Start: chr16: 17.76 End: chr16: 17.77	Mimida *et al.* (2015) Saito *et al.* (2013)	MDC020688.360 Scaffold317868 Start: chr15: 46.66 End: chr15: 46.67
8	Start: 11 End: 47	R_8450890_L8_36_1^a^SNP_ FB_0759171	Chr8: 7.50 Chr8: 23.30	Chr8: 8.2 Chr8: 27.0	MDP0000527190 (MdDAMd)	MCD008471.150 Start: chr8: 23.93 End: chr8: 23.95	Mimida *et al.* (2015) Saito *et al.* (2013)	
					MDP0000259294 (*MdDAMc*)	MDC020948.189 Start: chr8: 23.86 End: chr8: 23.87	Mimida *et al.* (2015) Saito *et al.* (2013)	

The information relating to the first version of the genome is available at the website http://www.rosaceae.org/ , whereas that of the third version was provided by the Foundazione Edmund Mach. Physical positions are in Mbp. All genes underlying major QTLs are avaible in Supplementary Table S3.

The second region of major interest is on LG12 (Fig. 4A–F), where QTLs were found for BB_CD and BB_GDH, which explained from 16.7% to 27.6% of the trait variance (Table 1). According to the Bayes factor, this LG carried one QTL for BB_CD in Mtp analysis and BB_GDH for multisite and Mtp analyses (Table 1). However, the QTL signal is spread over a large zone covering three possible peaks, until 67 cM. The trace plot (Fig. 4, left panel) indicates a switching signal between the last two peaks. There was evidence for the presence of MDP0000132050, also designated as *MdFT1*, just below the third peak of the QTL (Table 2). On LG7, there was a QTL mapped for BB_CD and BB_GDH (Fig. 4A–F) and which explains from 10.4% to 21.3% of the variance (Table 1). The QTL mapped on LG10 for BB_CD and BB_GDH (Fig. 4a–f) explained from 5.6% to 9.5% of the variance (Table 1). A weak but non-significant signal was found at that position for Delta_GDH (Fig. 4G–I).

Two additional regions were considered as important because *DAM* genes were predicted in the QTL interval. At the bottom of LG15, a QTL was detected for BB_CD and BB_GDH (Fig. 4; Table 1). In the third version of the genome, MDP0000322567, designated as *MdDAMa*, and a homolog to *AGL24* in *A. thaliana*, was predicted close to the beginning of the QTL (Table 2). This prediction is in contradiction to the prediction of the first version of the genome in which this gene was predicted on LG16 (Table 2). On LG8, a QTL was detected for BB_CD for multisite and Mpt analyses (Fig. 4A, B; Table 1). In this region, MDP0000259294 and MDP0000527190, designated as *MdDAMc* and *MdDAMd*, respectively, were predicted in the first version of the genome (Table 2).

Other QTLs were mapped in this study but were not detailed herein because of their low 2×lnBF values or their lack of stability across sites and families. In general, the QTLs mapped in the multisite analyses were also mapped in the Montpellier analyses, whereas there were fewer common QTLs between the multisite and the Angers analyses. Moreover, the 2×lnBF values were lower in Angers than in other analyses (Fig. 4).

### Year×genotype interaction-QTL mapping

Similarly to those observed for genotypic BLUPs, most QTLs for G×Y interaction BLUPs co-localized between multisite (Table 3) and Mtp analyses (Supplementary Table S1). Very few QTLs were detected for Ang analyses. QTLs mentioned in the previous section were also detected for interaction BLUPs in 2012 and 2014, especially on LG7, LG9, LG10, LG12, and LG15. However, new QTLs were detected on LG4 and LG11 for BB_CD in 2012 (Table 3). The QTL on LG4 was also detected for Delta_GDH in 2012. On LG8, a new QTL was detected for BB_CD in 2012. On LG15, three QTL regions were detected for Delta_GDH in 2012.

**Table 3:** Parameters associated with the interaction BLUP QTLs The first column indicates the type of analysis, the second one the trait concerned, and the third one the interaction BLUPs concerned. The following columns have the same meaning as in Table 1.^a^

**Analysis**	**Trait**	**BLUP**	**LG**	**2lnBF_LG**	**Max 2lnBF_loc**	**pos**	**add**	**fq**	**var**	**%var**
Multisite	BB_CD	Int 12	**4**	**10.1**	**7.4**	**27–37**	**0.45**	**0.46**	**0.101**	**14.5**
			**8**	**13.8**	**9.1**	**47–55**	**0.44**	**0.53**	**0.096**	**13.7**
			**11**	**5.4**	**8.1**	**7–17**	**0.45**	**0.41**	**0.096**	**13.7**
			**15**	**5.5**	**8.1**	**101–115**	**0.32**	**0.60**	**0.049**	**7.0**
		Int 14	4	2.9	4.6	9–25	0.18	0.34	0.015	5.4
			7	2.1	3.6	18–54	0.15	0.54	0.012	4.2
			9	3.9	7.5	2–10	0.22	0.41	0.024	8.7
	BB_GDH	Int 14	3	3.9	6.2	67–79	27.6	0.22	266	5.8
			5	2.7	5.9	14–24	29.8	0.33	393	8.6
			**7**	**12.7**	**8.3**	**54–60**	**38.6**	**0.27**	**585**	**12.7**
			**9**	**11.2**	**8.0**	**2–10**	**47.1**	**0.34**	**996**	**21.6**
			10	3.8	6.5	26–36	27.1	0.40	351	7.6
			**12**	**13.6**	**6.2**	**17–29**	**27.2**	**0.34**	**333**	**7.2**
	delta_GDH	Int 12	2	3.8	5.9	21–31	332.7	0.88	23 653	7.0
			**4**	**29.9**	**7.0**	**13–29**	**328.5**	**0.44**	**53 264**	**15.8**
			**15**	**10.6** ^***a***^	**14.1**	**1–3**	**684.8**	**0.10**	**83 240**	**24.6**
			**15**	**10.6** ^***a***^	**7.0**	**9–25**	**319.5**	**0.56**	**50 297**	**14.9**
			**15**	**10.6** ^***a***^	**8.4**	**59–67**	**383.2**	**0.89**	**29 065**	**8.6**
			**17**	**10.2**	**9.0**	**37–43**	**309.8**	**0.09**	**15 541**	**4.6**
		Int 14	**8**	**6.8**	**6.3**	**9–31**	**92.7**	**0.61**	**4096**	**14.0**
			**12**	**5**	**5.1**	**51–67**	**86.7**	**0.57**	**3684**	**12.6**
			15	3.2	6.8	7–17	100.2	0.67	4426	15.1
An	BB_CD	Int 12	4	3.8	5.0	29–59	0.41	0.41	0.08	26.1
			7	3.7	4.5	54–86	0.22	0.59	0.022	7.2
		Int 13	no QTL							
		Int 14	14	3.9	6.5	1–21	0.33	0.56	0.054	18.8
	BB_GDH	Int 12	no QTL							
		Int 13	no QTL							
		Int 14	no QTL							
	delta_GDH	Int 12	no QTL							
		**Int 13**	**3**	**6.9**	**6.3**	**65–87**	**126.5**	**0.41**	**7730**	**7.7**
		Int 14	no QTL							

^*a*^ 2×lnBF values for the comparison of a model with three QTLs to a model with two QTLs

### Estimated QTL genotypes

The estimated genotypes at each QTL can be used for three purposes: (i) to determine families that contributed to the discovery of QTLs by identifying the heterozygous parents; (ii) to identify the haplotype alleles that were linked in the coupling phase to the desired QTL alleles; and (iii) to evaluate the transmission of favorable alleles across the pedigree.

**Fig. 5.**
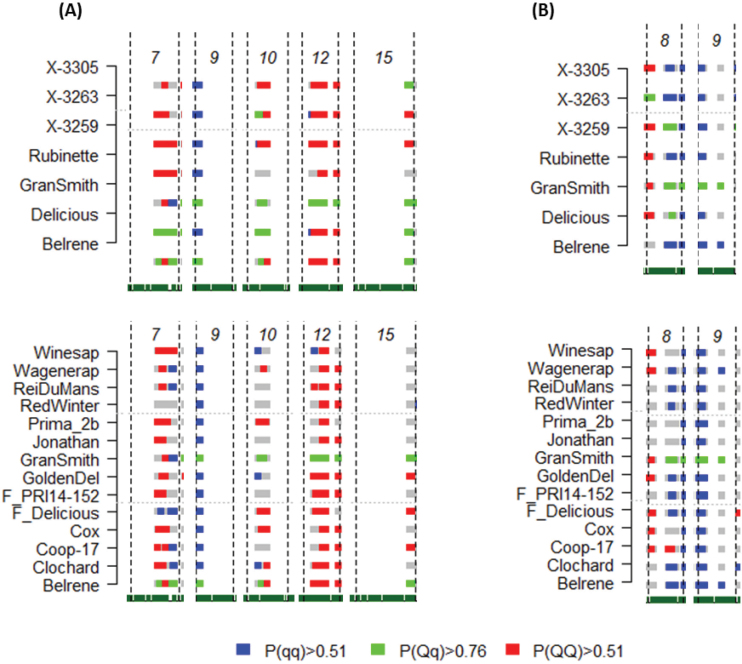
Posterior estimates of QTL genotype probabilities. Estimates are plotted for main regions for genotypic BLUPs for (a) BB_CD and (b) Delta_GDH. The beginning and the end of the chromosomes are represented by vertical dashed lines. Blue, green, and red colors indicate positive evidence for QTL genotypes qq, qQ, and QQ, respectively; q and Q refer to low and high phenotypic values, respectively. Gray colors indicate ignorance for a given genotype. Estimated genotypes of the parents and founders are presented, respectively. (This figure is available in colour at *JXB* online.)

For BB_CD, families SG and XB contributed the most to QTL detection. Indeed, the parents ‘Granny Smith’, ‘Delicious’, and ‘Belrene’ were estimated as heterozygous at most QTLs. The families HIVW, N, and P also contributed to some QTL segregation since the parents X-3263 and X-3305 were also estimated as heterozygous for QTLs on LG10 and LG15, respectively (Fig. 5A). However, the four progenitors of the Angers families, X-3305, X-3263, X-3259, and ‘Rubinette’, were estimated as homozygous for most QTLs.

For Delta_GDH, HIVW, N, and SG contributed the most to QTL mapping since X-3259 and ‘Granny Smith’ were estimated as heterozygous at most QTLs. XB also contributed since X3263 was estimated as heterozygous at some QTLs (Fig. 5B). Two progenitors of the Angers families, X-3305 and ‘Rubinette’, did not contribute to the detected QTLs since they were estimated as homozygous for all of the QTLs.

If we consider that favorable alleles are those that confer low values to BB_CD, most parents and founders were homozygous at the QTL on LG9 with the favorable allele, except ‘Granny Smith’ and ‘Belrene’, which were heterozygous. On LG15, parents or founders exhibiting favorable alleles were X-3305, ‘Granny Smith’, ‘Delicious’, and ‘Belrene’. X-3305 probably inherited the favorable allele from its paternal grandfather ‘Granny Smith’, while for ‘Delicious’, it was probably inherited from its parent ‘Winesap’ (Supplementary Figs S6, S7). Haplotype information is available in Supplementary Table S3. On LG7, X-3259 was homozygous with the favorable allele, and ‘Delicious’ and ‘Belrene’ were heterozygous. On LG10, only ‘Red Winter’ was identified as the homozygous parent with the favorable allele among parents and founders. On LG12, the uncertainty of the QTL position did not allow the identification of a favorable individual. A similar approach can be taken to identify favorable alleles for Delta_GDH (data not shown).

## Discussion

### Genetic determinism of budbreak and flowering time and QTL interpretation regarding CR and HR

The trait BB_CD was assumed to account for both HR and CR. In contrast, BB_GDH represented the variation of heat accumulation between the date of dormancy release estimated for ‘Golden Delicious’ and budbreak. Finally, Delta_GDH represented the heat accumulation between budbreak and flowering. Heritability of budbreak in calendar days (0.87) was much higher than in growing degree hours (0.38) and between flowering and budbreak (0.41). This suggests that the genotypic variation in CR may have a greater effect on genotype budbreak time than HR, as previously reported in several studies (Egea *et al.*, 2003; Ruiz *et al.*, 2007; Alburquerque *et al.*, 2008; Fan *et al.*, 2010; Celton *et al.*, 2011). The lower heritability of BB_GDH could also be due to a loss of variability in the computation. Indeed, the dormancy release date was estimated for ‘Golden Delicious’ for each site and year, and therefore did not account for a possible variability of dormancy release date among genotypes. For deciduous fruit trees, two methods have been employed to determine CR fulfillment. One is to expose cuttings harvested at different stages to a controlled warm condition for a period of time, with scoring of floral bud break (Gibson and Reighard, 2002). Another is to measure the weight of floral buds before and after exposure to warm conditions for a period of time (Tabuenca, 1964). Alternatively, the estimation of the dormancy release date from the budbreak date could be performed by modeling but would require long series of observations to determine the CR and HR of a genotype (Legave *et al.*, 2012).

Budbreak date in either CD or GDH shared four major QTLs on LG7, LG9, LG10, and LG12. This is consistent with the positive correlation between BLUPs of BB_CD and BB_GDH and suggests that the genetic control of these traits is partially shared. However, other chromosomal regions were detected, suggesting a multigenic control.

Four detected regions could result from chilling perception since they co-localized with candidate genes linked to this mechanism. Under the major QTL at the top of LG9, MDP0000126259, an *FLC*-like gene, was predicted in the first version of the genome. This gene has been shown to be differentially expressed in a CR study, supporting a role in repression of bud growth during ecodormancy (Porto *et al.*, 2015). Close to the same region in both the first and third versions of the apple genome, MDP0000143531, which is a homolog to *AGL24*, a *MADS-box* transcription factor belonging to the same gene family as *DAM* genes, was also predicted (Yamane *et al.*, 2011; Wells *et al.*, 2015). Since *AGL24* is a floral promoter regulated by an *FLC*-independent vernalization pathway (Michaels *et al.*, 2003), this suggests complementary roles of these genes in chilling perception and endodormancy regulation. Two other QTLs, on LG15 and LG8, co-localized with three of the four *DAM* genes annotated in the apple genome which constitute a cluster of genes unique to *Rosaceae* that play a role in the establishment of endodormancy and its maintenance (Mimida *et al.*, 2015). These *DAM* genes were predicted on LG8, *MdDAMc* and *MdDAMd*, and on LG15, *MdDAMa.* The prediction of *MdDAMa* on LG15 in the third version of the apple genome, instead of LG16 in the first version, is more consistent with both LG8 and LG15 homology (Velasco *et al.*, 2010) and the present QTL detection. Finally, at the bottom of LG12, an *FT* homolog was mapped, such as in Guitton *et al.* (2011). In perennial species such as *Populus*, *FT* homologs have been shown to be linked to growth cessation in response to winter temperature, photoperiod, and dormancy establishment (Böhlenius *et al.*, 2006; Kotoda *et al.*, 2010; Tränkner *et al.*, 2010; Hsu *et al.*, 2011; Srinivasan *et al.*, 2012). This constitutes a new and complementary assumption for dormancy regulation in apple.

Regarding the perception of warm temperatures after budbreak, represented by Delta_GDH, a QTL was also detected at the top of LG9 despite a weak signal. Since growth resumption after CR fulfillment and under favorable temperatures results from the capacity of cells to divide and elongate (Rohde and Bhalerao, 2007), genes involved in the regulation of cell cycles or associated with plant hormones have been identified under QTLs associated with budbreak, particularly at the top of LG9 (Celton *et al.*, 2011). In this study, we focused on candidate genes related to chill perception, but the high stability of the QTL at the top of LG9 across studies (Dyk *et al.*, 2010; Celton *et al.*, 2011) and the candidate genes predicted in the same region suggest that it could contain a cluster of key genes involved in chill perception and cell cycle reactivation. This is consistent with the conclusion of Wisniewski *et al.* (2015) that dormancy, growth regulation, and budbreak are likely to be interdependent mechanisms.

### Contribution of QTLs to specific phenological stages or climatic conditions

In this study, differences in temperatures between years and sites were taken into account in the computation of GDH traits and fixed effects in mixed linear models. Regions detected for genotype×year interaction BLUPs were generally similar to those detected for genotypic BLUPs, suggesting that specific temperature reinforced the effect of one or several QTLs. However, new regions were specifically detected in 2012 and 2014 on LG4, LG8, LG11, and LG15. Since 2012 was characterized by an extended time required to release dormancy for ‘Golden Delicious’ in Montpellier (Fig. 2), warm winter conditions could enhance the effect of known regions such as the top of LG9 and trigger other regions. Finally, as suggested by Celton *et al.* (2011), minor QTLs could contribute to additional genetic effects in specific years and climatic conditions for specific stages.

The wider variability of phenotypes in Montpellier compared with Angers can be due to differences between parents, operators, or climatic conditions. Consequently, the interaction between site and genotype could not be estimated. Even though CR and HR were unknown for most parents, it is likely that they have different requirements. Also, despite the same protocol used between the two sites, phenotyping was carried out by different operators, possibly leading to specific effects that could not be distinguished from the site effect. Finally, at Mediterranean sites, dormancy release dates are more impacted by changing temperatures than in cooler climates (Legave *et al.*, 2012). However, the high CR cultivar ‘Golden Delicious’ was used in these estimations and lower CR cultivars could be less impacted by climate differences. Since most QTLs detected for multisite analysis were common with Mtp analysis, the climatic conditions in Montpellier could be more suitable to reveal differences in CR among progeny because of a larger variability in the time to CR fulfillment than in Angers.

### Comparison of QTL analysis between single family and multifamily studies

Compared with previous studies (Dyk *et al.*, 2010; Celton *et al.*, 2011), more QTLs were detected, among which the QTL at the top of LG9 was common whereas QTLs on LG7, LG10, and LG12 were newly revealed regions. This confirms the expected advantages of multipopulation studies (Bink, 2002; Blanc *et al.*, 2006; Liu *et al.*, 2012). However, the percentage of variance explained by most QTLs was lower in this study than in previous studies, whereas the total variance explained by all the QTLs was higher for most traits. This could be due to genetic variability spread over more genomic regions. Finally, the size of QTL intervals was similar between studies, suggesting that the QTL position, for example on LG9, could be precisely estimated in single family studies, mainly due to the number of individuals considered (Collard *et al.*, 2005).

### Contribution of families and breeding perspectives

The families that segregate at major QTLs for BB_CD and BB_GDH are SG, XB, and HIVW, whereas SG, XB, N, and P segregate for Delta_GDH. In addition, N and P families that segregate for Delta_GDH could also provide interesting individuals for the variation in heat perception. Breeding for low CR will certainly be more crucial for Mediterranean and subtropical climates than for temperate climates since low chill cultivars could be one solution to avoid dormancy breaking problems (Campoy *et al.*, 2011). Parents ‘Granny Smith’, ‘Belrène’, X-3305, and ‘Delicious’ displayed favorable alleles, therefore their descendants are expected to provide interesting progenitors possibly bearing and cumulating the favorable alleles in single individuals. Since low HR may lead to early budbreak and bud damage due to spring frost in either warm or cold climates, the recommendation would therefore be to avoid low HR varieties whatever the climate. In addition, high HR could be difficult to fulfill in cold climates and should be avoided as well. In any case, HR could be used to adjust budbreak timing to other constraints such as the phenology of pollinator varieties or insects. In the present study, the small size of several families was compensated by relatedness between families due to a common parent.

In spite of major QTLs detected for budbreak timing, we demonstrated the highly polygenic control of budbreak. In a breeding context, many small effect QTLs could contribute to an increase in the total variance explained and the prediction robustness. In this perspective, genome-wide selection models will be a complementary approach to QTL analysis in order to evaluate the genetic value of individuals by summing up allelic effects along the genome, provided that the marker density is high enough (Jannink *et al.*, 2010).

## Supplementary data

Supplementary data are available at *JXB* online.

Table S1 Parameters associated with the interaction BLUP QTLs for the Montpellier analysis.

Table S2 Haplotype composition for the QTL located on LG15 between 105 cM and 107 cM.

Table S3 List of genes underlying major QTLs in the first and third versions of the apple genome.

Figure S1 Proportion of informative meiosis for haploblock markers along the genome.

Figure S2 Daily temperature during winter 2011–2012 and spring 2012.

Figure S3 Daily temperature during winter 2012–2013 and spring 2013.

Figure S4 Daily temperature during winter 2013–2014 and spring 2014.

Figure S5 Correlation plot and *r* value between genotypic BLUP for BB_CD and BB_GDH

Figure S6 Estimated genotypes and corresponding haplotypes for QTL located on LG15 between 105 cM and 111 cM of the SG family and its progenitors.

Figure S7 Estimated genotypes and corresponding haplotypes for the QTL located on LG15 between 105 cM and 111 cM of the P family and its progenitors.

Supplementary Data

## Abbreviations:

Ang: analysis with only families grown in Angers
BB: budbreak date
BB_CD: budbreak date expressed in CD
BF: beginning of flowering date
BV: breeding value
CD: calendar days
CR: chilling requirement
delta_GDH: time between BF and BB expressed in GDH
GBV: genome-wide breeding value
GDH: growing degree hours
HIVW: full-sib family derived from the cross between X-3263 and X-3259
HR: heat requirement
Mtp: analysis with only families grown in Montpellier
multisite: analysis with full data set containing the five families
N: full-sib family derived from the cross between X-3305 and X-3259
P: full-sib family derived from the cross between ‘Rubinette’ and X-3305
SG: full-sib family derived from the cross between ‘Starkrimson’ and ‘Granny Smith’
XB: full-sib family derived from the cross between X-3263 and ‘Belrene’
