# Supplementary material for: Detecting QTLs and putative candidate genes involved in budbreak and flowering time in an apple multiparental population
Source: J Exp Bot. 2016 Mar 31;67(9):2875–88. doi: 10.1093/jxb/erw130 (PMC4861029; doi:10.1093/jxb/erw130)
Supplement: Supplementary Data [file supp_67_9_2875__index.html]

Detecting QTLs and putative candidate genes involved in budbreak and flowering time in an apple multiparental population — Detecting QTLs and putative candidate genes involved in budbreak and flowering time in an apple multiparental population — Supplementary Data 

# Detecting QTLs and putative candidate genes involved in budbreak and flowering time in an apple multiparental population

## Supplementary Data

Data files

- supplementary\_figures\_S1\_S7\_tables\_S1\_S2.pdf - Supplementary Data
- supplementary\_table\_S3.xlsx - Supplementary Data
