# Supplementary material for: Detecting QTLs and putative candidate genes involved in budbreak and flowering time in an apple multiparental population
Source: J Exp Bot. 2016 Mar 31;67(9):2875–88. doi: 10.1093/jxb/erw130 (PMC4861029; doi:10.1093/jxb/erw130)
Supplement: Supplementary Data [file supp_erw130_supplementary_figures_S1_S7_tables_S1_S2.pdf]

Detecting QTL and putative candidate genes involved in budbreak and flowering time in an apple multi-parental population

A. Allard, J.M. Legave, S. Martinez, J.J. Kelner, M.C.A.M. Bink, M. di Guardo, E.A. Di Pierro, F. Laurens, W.E. van de Weg, E. Costes

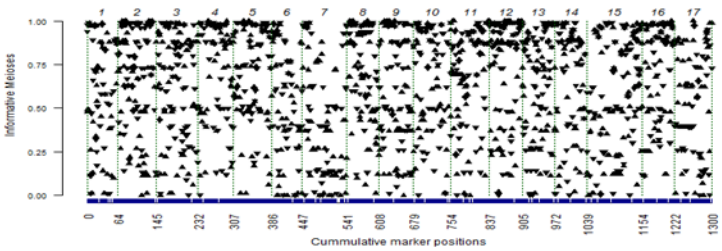

Figure S1: Proportion of informative meiosis for haploblock markers along the genome. On the X-axis, the beginning and the end of the chromosomes are represented by vertical dashed lines.

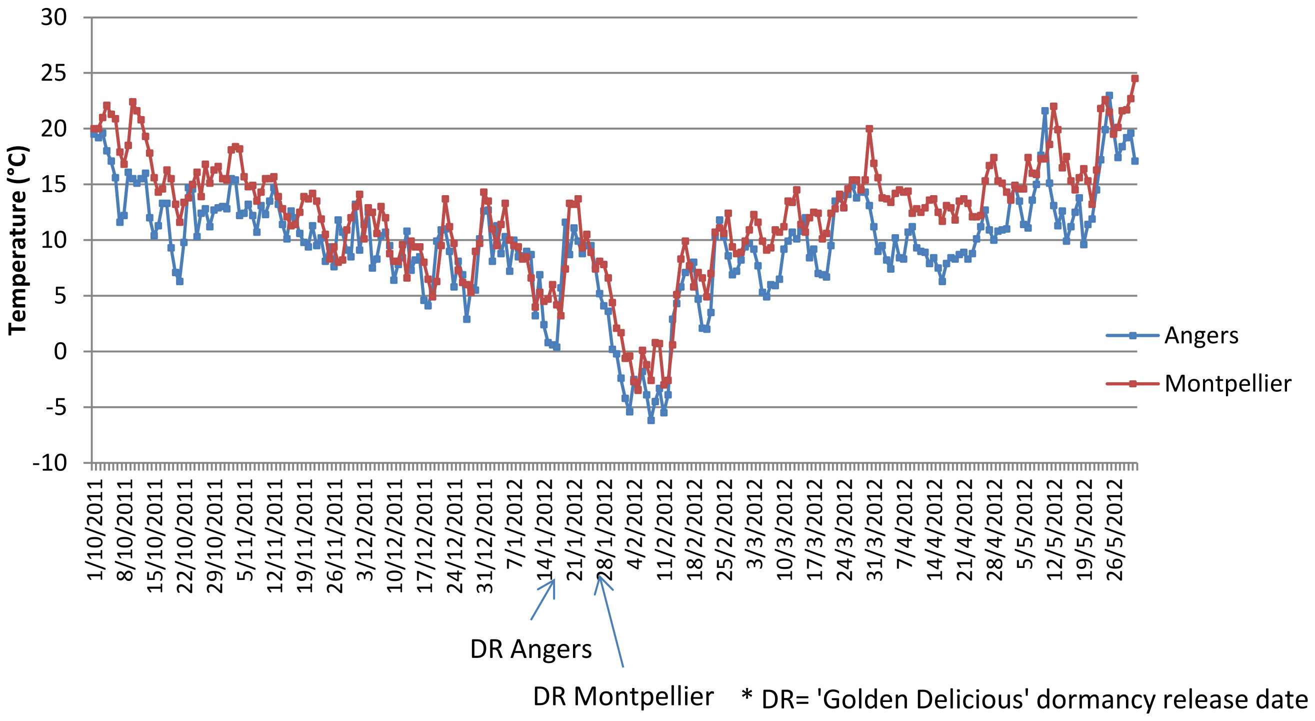

Figure S2: Daily temperature during chilling and heat fulfilment in winter 2011-2012 and spring 2012.

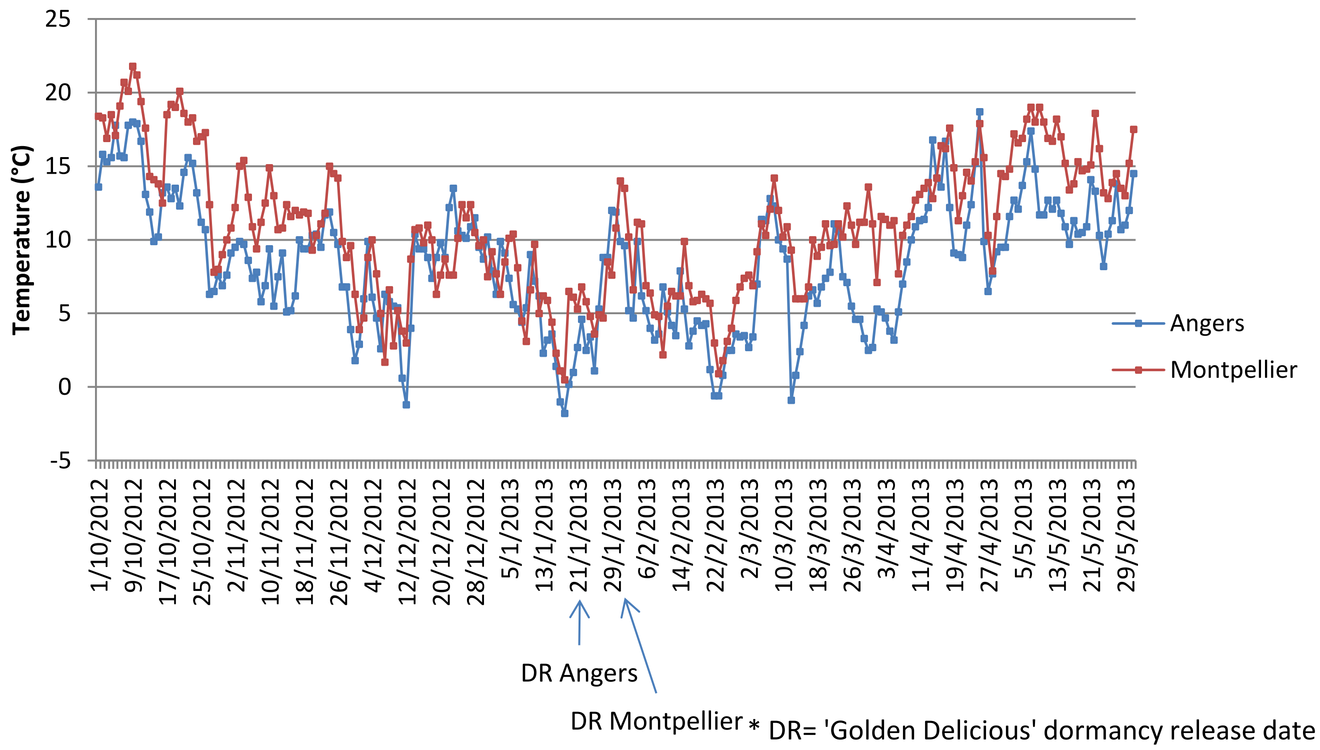

Figure S3: Daily temperature during chilling and heat fulfilment in winter 2012-2013 and spring 2013.

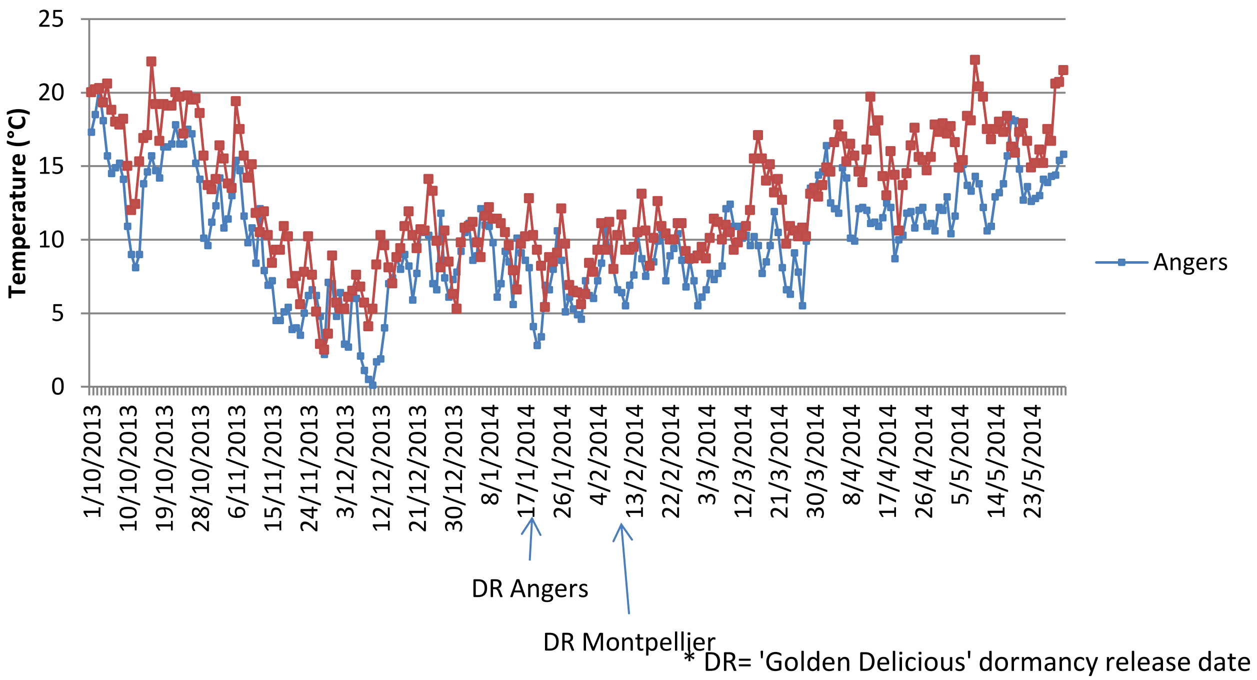

Figure S4: Daily temperature during chilling and heat fulfilment in winter 2013-2014 and spring 2014.

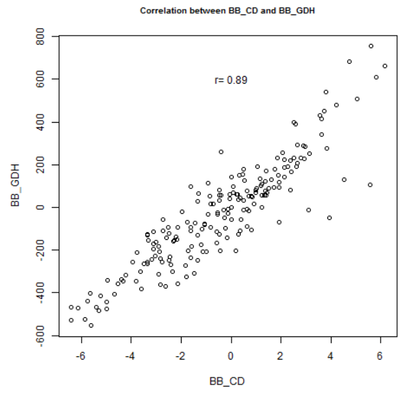

Figure S5: Correlation plot and r value between genotypic BLUP for BB_CD and BB_GDH

Table S1: Parameters associated with the interaction BLUP QTLs for the Montpellier analysis. The first column indicates the type of analysis, the second one the trait concerned, and the following columns indicate the LG where the QTL is located, the 2*lnBF value at the LG scale for a 1 QTL over a 0 QTL model, the 2*lnBF value at the local scale, the position of the QTL region in cM, the additive QTL effect, the frequency of the positive allele, its variance and its percentage of variance explained. The 2*lnBF values for multi-QTL models were not presented because none of them passed the significance threshold. The QTLs with a 2*lnBF at the LG scale higher than 5 are in bold type.

Analysis	Trait	LG	2lnBF_LG	Max 2lnBF_loc	Pos (cM)	add	fq	var	%var
BB_CD	int 12	4	4.3	5.5	11-35	0.52	0.52	0.13	13.8
		**8**	**6.0**	**5.8**	**39-65**	**0.43**	**0.54**	**0.09**	**9.2**
		12	3.1	3.9	39-61	0.37	0.49	0.07	7.0
		15	2.3	5.3	101-115	0.42	0.62	0.08	8.3
	int 13	4	2.8	4.7	1-15	0.76	0.44	0.28	8.6
		14	4.3	6.8	45-65	1.00	0.46	0.49	15.0
	int 14	3	2.5	4.6	59-79	0.27	0.47	0.04	10.3
		7	3.2	4.0	14-40	0.26	0.55	0.03	9.2
		9	3.1	5.5	2-14	0.27	0.50	0.04	10.4
BB_GDH	int 12	8	4.8	5.3	1-37	338.2	0.47	56921	8.3
		12	4.4	5.0	33-67	360.1	0.48	64681	9.4
		15	3.3	5.7	97-109	349.9	0.60	58620	8.5
	int 13	**7**	**6.8**	**5.7**	**58-86**	**204.5**	**0.44**	**20573**	**9.2**
		8	2.9	4.7	15-45	162.0	0.49	13127	5.9
		**9**	**10.3**	**8.1**	**2-12**	**256.4**	**0.53**	**32756**	**14.7**
		**10**	**5.9**	**8.4**	**26-34**	**182.4**	**0.62**	**15753**	**7.1**
		**12**	**11.3**	**6.2**	**19-45**	**245.3**	**0.53**	**29951**	**13.4**
		**14**	**5.3**	**6.7**	**43-55**	**278.1**	**0.33**	**34392**	**15.4**
	int 14	3	2.4	4.3	59-85	28.3	0.46	398	0.2
		**7**	**5.3**	**6.4**	**52-64**	**36.9**	**0.55**	**675**	**12.6**
		9	4.3	6.5	2-12	39.2	0.50	767	14.4
		**12**	**5.3**	**6.2**	**11-43**	**32.0**	**0.54**	**507**	**9.5**
delta_GDH	int 12	15	3.8	5.1	53-77	247.4	0.46	30392	11.8
	int 14	**8**	**4.7**	**5.9**	**11-31**	**102.5**	**0.45**	**5197**	**13.4**
		12	3.3	5.4	53-67	90.4	0.51	4089	10.5
		15	2.6	4.9	7-23	101.4	0.54	5105	13.1

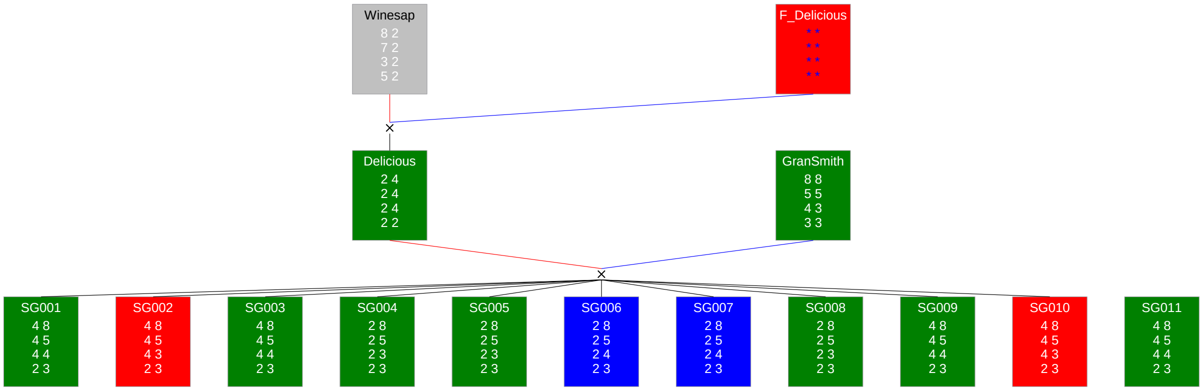

Figure S6: Estimated genotypes and corresponding haplotypes for a QTL located on LG15 between 105 and 111 cM of the SG family and its progenitors. The name of the individual and the alleles for four successive haploblocks located at 107, 108, 109 and 110 cM, respectively, on LG15 are given in each box. The color of the boxes indicates the estimated genotypes at the QTL. Red and blue boxes correspond to homozygous individuals with alleles for high (QQ) and low (qq) genotypic values, respectively, green boxes correspond to heterozygous individuals, and gray boxes to individuals for which no distinction could be made between heterozygosity and homozygosity.

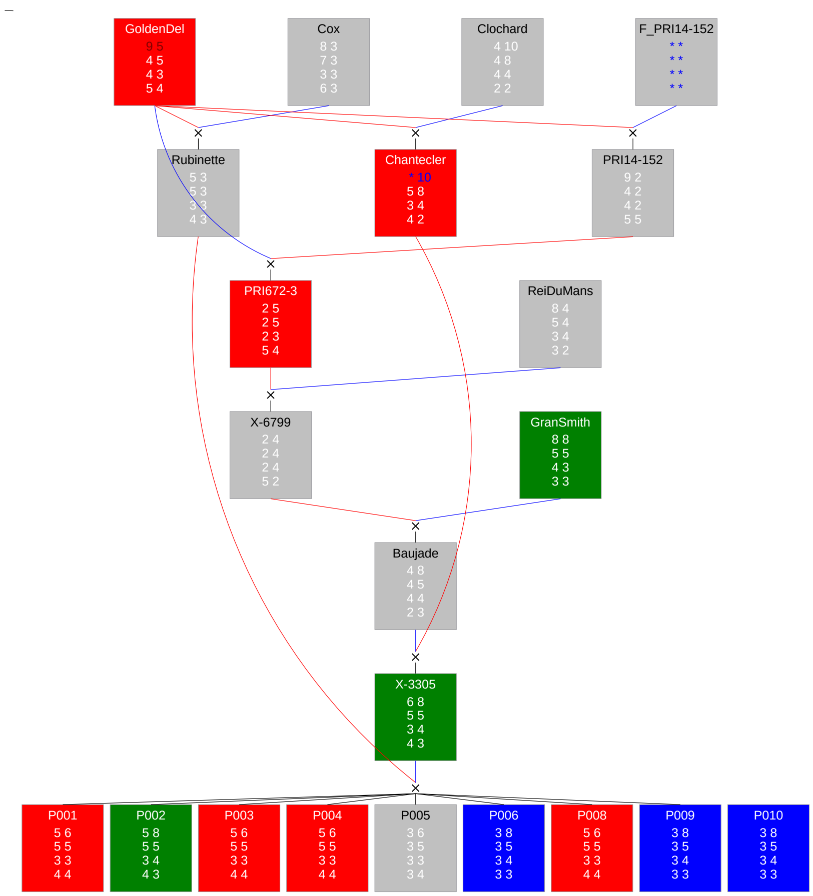

Figure S7: Estimated genotypes and corresponding haplotypes for a QTL located on LG15 between 105 and 111 cM of the P family and its progenitors. For the legend, see Fig. S3.

Table S2: Indication of haplotype composition for the QTL located on LG15 between 105 and 107 cM. The first column indicates the haplotype name, the second its position on LG15 and the number of SNP markers composing it, the third the haplotype allele ID, the fourth the frequency of the allele, and the following columns the composition of the haplotype allele at each marker. The names of the markers are given in the last column. Missing values are indicated by a '-' symbol.

Haplotype	Position (cM)	Marker_nb	Haplo_ID	freq	M1	M2	M3	M4	M5	M6	M7	M8		
		8	1	9	-	-	-	-	-	-	-	-	M1:	F_0323470_L15_PA
		8	2	89	1	2	1	1	2	2	2	1	M2:	F_0323476_L15_PA
		8	3	58	1	1	2	1	2	2	1	2	M3:	F_0323478_L15_PA
		8	4	185	1	2	1	2	1	1	2	2	M4:	F_0323485_L15_PA
		8	5	26	1	2	2	1	2	2	1	2	M5:	F_0323486_L15_PA
		8	6	154	2	2	1	1	1	2	1	2	M6:	F_0324449_L15_PA
		8	7	122	1	2	2	1	2	2	2	2	M7:	F_0324471_L15_PA
		8	8	285	1	2	1	1	2	2	1	2	M8:	F_0324474_L15_PA
		8	9	2	2	2	1	1	1	2	2	1		
		8	10	2	2	2	1	1	1	1	2	2		
fp15_107	107	8	11	0	1	-	1	1	2	2	1	2		
		8	12	0	1	2	1	2	1	2	1	2		
		8	13	0	1	-	1	2	1	1	2	2		
		8	14	0	1	-	2	1	2	2	1	2		
		8	15	0	1	2	1	1	2	2	-	2		
		8	16	0	2	2	1	1	1	2	-	2		
		8	17	0	1	2	1	2	1	1	-	2		
		8	18	0	1	2	2	1	2	2	-	2		
		8	19	0	1	2	1	2	-	1	-	2		
		8	20	0	2	2	1	1	-	2	-	2		
		8	21	0	1	2	1	2	2	1	-	2		
		8	22	0	1	2	1	2	1	1	2	1		
		5	1	5	-	-	-	-	-				M1:	F_0323965_L15_PA
		5	2	88	2	2	1	1	1				M2:	F_0323976_L15_PA
		5	3	59	2	1	2	2	2				M3:	F_0323978_L15_PA
fp15_108	108	5	4	190	1	2	2	1	1				M4:	F_0323993_L15_PA
		5	5	344	2	1	1	1	2				M5:	F_0323994_L15_PA
		5	6	2	2	2	2	1	2					
		5	7	242	2	1	2	1	2					
		5	8	2	2	1	2	1	1					
		2	1	6	-	-							M1:	F_0042270_L10_PA
		2	2	90	2	2							M2:	F_0042295_L10_PA
fp15_109	109	2	3	547	1	1								
		2	4	289	1	2								
		2	5	0	1	-								
		2	6	0	2	-								
		4	1	5	-	-	-	-					M1:	F_0915567_L15_76_1
		4	2	275	1	1	2	1					M2:	F_0915575_L15_76_1
		4	3	226	2	2	2	1					M3:	F_0915601_L15_76_1
fp15_110	110	4	4	179	2	2	1	2					M4:	F_0326218_L15_PA
		4	5	125	1	2	2	1						
		4	6	1	2	2	2	2						
		4	7	121	2	2	1	1						

Table S3: List of genes underlying major QTLs in first and third versions of the apple genome. Each tab contains the list of genes underlying a QTL, indicated in the name of the tab. In each tab, the columns with green headlines contain gene information on the third version of the genome: gene ID, contig ID, position and length of the contig, chromosome, scaffold. Columns with gray headlines contain gene information on the first version of apple genome and homolog genes in *Arabidopsis thaliana* and their annotations.
